# Association between air pollution and suicide: a time series analysis in four Colombian cities

**DOI:** 10.1186/s12940-018-0390-1

**Published:** 2018-05-12

**Authors:** Julián Alfredo Fernández-Niño, Claudia Iveth Astudillo-García, Laura Andrea Rodríguez-Villamizar, Víctor Alfonso Florez-Garcia

**Affiliations:** 1 Departamento de Salud Pública, Universidad del Norte, Barranquilla, Colombia; 2 Servicios de Atención Psiquiátrica, Secretaría de Salud, Ciudad de México, Mexico; 3 Departamento de Salud Pública, Universidad Industrial de Santander, Bucaramanga, Colombia

**Keywords:** Suicide, Air pollution, Confounding factors, Epidemiology

## Abstract

**Background:**

Recent epidemiological studies have suggested that air pollution could be associated with suicide. However, other studies have criticized these results for being analytically weak and not taking into account potential confounding factors. As such, further studies examining the relationship under diverse contexts are necessary to help clarify this issue. This study explored the association between specific air pollutants (NO_2_, SO_2_, PM_10_, PM_2.5_, CO and O_3_) and suicide incidence in four Colombian cities after adjusting for climatic variables and holidays.

**Methods:**

A time series of daily suicides among men and women living in Bogota, Medellin, Cali and Bucaramanga was generated using information from the National Administrative Department of Statistics (DANE) for the years 2011–2014. At the same time, the average daily concentration of each air pollutant for each city was obtained from monitoring stations belonging to the National Air Quality Surveillance System. Using this information together, we generated conditional Poisson models (stratified by day, month and year) for the suicide rate in men and women, with air pollutants as the principal explanatory variable. These models were adjusted for temperature, relative humidity, precipitation and holidays.

**Results:**

No association was found between any of the examined pollutants and suicide: NO_2_ (IRR:0.99, 95% CI: 0.95–1.04), SO_2_ (IRR:0.99, 95% CI: 0.98–1.01), PM_10_ (IRR:0.99, 95% CI:0.95–1.03), PM_2.5_ (IRR:1.01, 95% CI: 0.98–1.05), CO (IRR:1.00, 95% CI:1.00–1.00) and O_3_ (IRR: 1.00, 95% CI: 0.96–1.04). In the same way, no association was found in stratified models by sex and age group neither in lagged and cumulative effects models.

**Conclusions:**

After adjusting for major confounding factors, we found no statistically significant association between air pollution and suicide in Colombia. These “negative” results provide further insight into the current discussion regarding the existence of such a relationship.

## Background

In 2015, approximately 788,000 individuals committed suicide worldwide, which is equivalent to an age-standardized rate of 10.7 individuals per 100,000 inhabitants [1]. In the 15–29 year age group, suicide was the second leading cause of death [2]. As such, suicide currently poses a very important public health problem, especially in developing countries where the rate is increasing [1].

In Colombia, there were 24,882 suicides between 2000 and 2010, which means an average of 6.2 per day that increases up to 8.0 per day in holidays. Despite the suicide rates have shown a downward trend since 2000, suicides remain a major challenge, as 50% of them are committed by people under 31 years old [3].

Suicide is a very complex and multifactorial event, with risk factors existing at both the individual and contextual level [2]. Since the nineteenth century, the effect of environmental determinants on suicide has been widely investigated [4, 5]. For instance, meteorological factors such as rain, increases in temperature and drought, have been closely related with seasonal behaviors of suicide in certain countries [6, 7]. More recently, environmental pollution, specifically air pollution, has been proposed as another determinant of suicide incidence at the ecological level [8–12].

Although the harmful effects of air pollution are mainly associated with respiratory and cardiovascular diseases [13], more recent evidence also points towards adverse effects for the central nervous system. According to the hypothesis of neuroinflammation, air contaminants could cause an increase in cytokines and reactive oxygen species, and consequently self-aggressive behavior [14, 15]. The causal mechanisms between air pollution and suicide might not only involve this direct pathway via inflammation, but also the exacerbation of mental disorders that increase the risk of suicidal behavior [16, 17]. Nonetheless, these pathways are not yet clearly established, and are far from being biologically proven.

Due to poor methodological support and possible confounding factors, the epidemiological associations between air pollution and suicide are quite controversial and inconclusive [18–20]. As such, it is important not only to further explore this relationship in other socio-environmental contexts, but also to adjust for the main confounding factors. For instance, as shown in several studies, climate itself can be associated with pollution levels [18–20]. Furthermore, as in other air quality and health studies, the use of appropriate statistical techniques is highly important to control for seasonality and autocorrelation of observations [19, 21].

A recent systematic review suggests a certain consistency in the association between air pollution and suicide [22]. However, the conclusions of this review should be taken with caution because when a new epidemiological association is reported, it is well known that there is a tendency to first publish the “positive” results. This leads to a publication bias and as such, an undesirable impact on the conclusions of systematic reviews and meta-analyses [23]. Thus, the replication and publication of rigorous studies, even those with “negative” results, enables clarification of the relationships and avoids potential biases.

This study explored the relationship between air pollution and suicide incidence in four capital cities of Colombia. Specifically, we consider the mass concentrations of nitrogen dioxide (NO_2_), sulfur dioxide (SO_2_), carbon monoxide (CO), ozone (O_3_), and particulate material with aerodynamic diameters less than 10 and 2.5 μm (PM_10_, and PM_2.5_, respectively), and adjusted our results according to weather conditions and holidays.

## Methods

### Study type and observation units

We conducted a multi-city ecological time series study in which the unit of observation was city/day. We included four of the five main capital cities of Colombia (Bogota, Bucaramanga, Cali, and Medellin) because they had daily air pollutant measurements available for the entire four-year study period (January 1, 2011 to December 31, 2014).

### Variables and data sources

#### Outcome: suicide counts

For each city, the number of suicides per day were calculated taking into account the following ICD-10 codes as the principal cause of death: X60-X84 and Y87.0. This data was obtained from the mortality database of the National Department of Statistics (DANE) for the indicated study period. The total population of each city, which was obtained as a projection based on DANE data for each year, was considered as the exposure variable in our analyses. As the total population of a city hardly changes over the course of a year, our analyses enable comparisons of the suicide counts between years, and more importantly, between cities.

#### Exposure variable: air pollution

Daily data from the National Air Quality Surveillance System was obtained for the following air pollutants: CO, NO_2_, SO_2_, O_3_, PM_10_ and PM_2.5_. These measurements were obtained from the air quality monitoring stations located across the four cities: 13 in Bogota, 7 in Medellin, 5 in Cali and 4 in Bucaramanga. For each city, we calculated the daily averages for NO_2_, PM_10_, PM_2.5_, and SO_2_, and the maximum 8-h moving averages for CO and O_3_, by averaging information from all monitoring stations.

#### Confounders

The main confounding factors were meteorological variables and holidays. Daily meteorological data regarding temperature (in degrees Celsius), relative humidity (%), and precipitation (in mm) were obtained from 19 meteorological stations (11 in Bogota, 4 in Medellin, 2 in Cali, and 2 in Bucaramanga) located in different points across the four cities. This information was obtained from the Institute of Hydrology, Meteorology and Environmental Studies (IDEAM, by its initials in Spanish). For the analysis of holidays, we generated an indicator variable as follows: ordinary day (reference category), holiday, and long weekend (this last one refers to a Colombia tradition that there are weekends with non-working Mondays given by the relocation of some holidays). As holidays change according to the year, historical calendars were used to retrieve the relevant information.

### Statistical analysis

We generated a description of the daily suicide rates by city. By using the dispersion index (VIT) [24] and the asymptotic Böhning test [25], we verified the equidispersion assumption (i.e., the null hypothesis of a Poisson distribution could not be rejected; *p* > 0.20). For each time series, a Dickey Fuller test was performed to explore the existence of a unitary root, which is typical of a non-stationary model.

The association between air quality and suicides was explored by using a multi-city Poisson model conditioned by time strata (grouping by day, month and year) to control for the seasonality of suicide data. In this way, effects are estimated considering the structure of the correlation that the observations would have when they are generated on the same stratum of day of the week, month, and year [26], with the city as an indicator variable by using the following fixed effect model:

$$ \mathrm{Ln}\left({\mathrm{Y}}_{\mathrm{i},\mathrm{s}}\right)={\mathrm{B}\mathrm{o}}_{\mathrm{s}}+{\mathrm{B}}_1{\mathrm{X}}_{1\mathrm{i}}+{\mathrm{B}}_2{\mathrm{X}}_{2\mathrm{i}}+{\mathrm{B}}_3{\mathrm{X}}_{3\mathrm{i}}+{\mathrm{B}}_4{\mathrm{X}}_{4\mathrm{i}}+{\mathrm{B}}_5{\mathrm{X}}_{5\mathrm{i}}+{\mathrm{B}}_6{\mathrm{X}}_6+\mathrm{Ln}\left(\uplambda \right),\mathrm{Y}\approx \mathrm{Poisson}\left({\upmu}_{\mathrm{i}}\right) $$

Where Y_i,s_ is the suicide count in the day i that falls in stratum s, X is the vector of independent variables: X_1_ air pollutant; X_2_ temperature X_3_ relative humidity, X_4_ precipitation, X_5_ holidays indicador, X_6_ city indicator, and λ is the exposure variable (population) with coefficient forced to be 1.

These conditional time series models allow for adequate control of the seasonality of the variables under analysis. Moreover, they require lower computational intensity compared to other time series models, and yield similar estimation results to those that would be obtained using a case-crossover model for analysis of individuals [27].

In order to facilitate interpretation, the daily concentrations of the pollutants were centered by the integer value that approximately corresponds to 20% of the average concentration of each pollutant in the time series. With respect to PM_10_ and PM_2.5_, the values were centered by convention on 10 and 5 μg/m^3^, respectively. Models were stratified by sex and age group (Children under 15 years old, adults from 15 to 60 years and older adults aged 60 years and over). Finally, with the objective of exploring potential lagged effects, single lagged effects were explored from 1 up to 7 days and cumulative effects were explored for moving average estimates of lagged days 1 to 7 by using fixed models at city level without conditioning by time strata. Fixed models were chosen at the city level since, when performing the Hausman test, slight systematic differences were found between the estimators obtained from the fixed effects model and those estimated using random effects for the city. This decision was made given that the estimates of fixed effects are more unbiased than those of random effects.

Associations were considered statistically significant at an alpha of 0.05 with a Bonferroni correction given as α/m where m is the number of hypothesis in each analysis. All models were adjusted by temperature, relative humidity and holidays. Models were evaluated using the distribution of residuals and the goodness of fit tests. All analyses were performed using STATA 14 (Stata Corporation, College Station, TX, USA) (26).

## Results

**Fig. 1:**
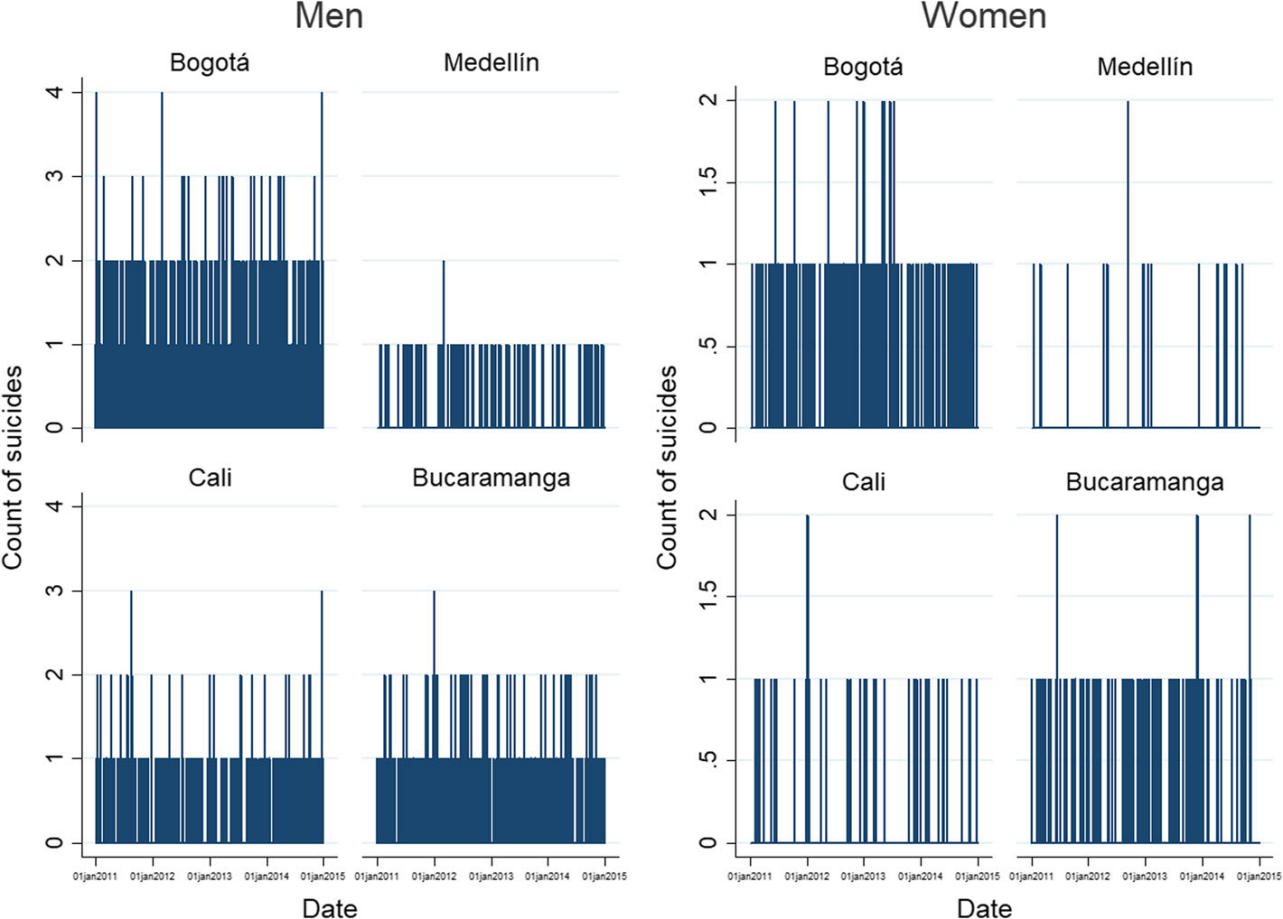
Daily suicide counts by sex in four Colombian cities, 2011–2014

Over the entire study period, there were a total of 1942 suicides (average of 1.37 per day) in the four cities: 1000 suicides occurred in Bogota (808 of whom were males), 133 in Medellin (95 males), 311 in Cali (266 males) and 498 in Bucaramanga (387 males). Figure 1 shows the daily number of suicides over the entire study period by sex and city. In Bogota, the average number of suicides per day was 0.68 (95% CI: 0.64–0.73), with a variance of 0.72. Medellin on the other hand, was the city with the lowest daily average number of suicides and variance, with values of 0.08 (95% CI: 0.06–0.09) and 0.08, respectively. In the case of Cali, the daily average number of suicides was estimated at 0.21 (95% CI: 0.19–0.24), with a variance of 0.22. Finally, the average daily suicide counts for Bucaramanga was 0.34 (95% CI: 0.31–0,37), with a variance of 0.32.

In all cities, the low daily averages can be attributed to the high proportion of days in which zero suicides were registered. In fact, of the 1461 days considered in this study, 51.47, 92.47, 81.11, and 70.70% of them had no suicides in Bogotá, Medellin, Cali, and Bucaramanga, respectively. Using equi-dispersion tests, we proved that the behavior of the daily counts for each city were as expected according to a Poisson distribution. The Dickey-Fuller test rejected the unit root hypothesis, thereby suggesting a stationary behavior in all four cities. Descriptive data for air pollutants concentrations and meteorological data by city is presented in Table 1.

**Table 1:** Air pollutant concentrations and meteorological conditions in four Colombian cities, 2011–2014

	Bogota				Medellin				Cali				Bucaramanga			
Variables/City	Mean (SD)	P10	P90	Min/Max	Mean (SD)	P10	P90	Min/Max	Mean (SD)	P10	P90	Min/Max	Mean (SD)	P10	P90	Min/Max
CO (mg/m^3^)	1.38 (0.41)	0.9	1.88	0.42/3.33	2.7 (1.05)	1.7	4	1/6.2	1.75 (0.58)	0.95	2.5	0.3/3.45	2.07 (0.62)	1.3	2.87	0.55/4.1
NO_2_ (μg/m^3^)	32.35 (8.04)	22.54	42.88	12.27/62.5	37.84 (9.93)	25.83	50.57	10.87/107.2	14.45 (4.53)	8.6	19.6	2.8/37.3	23.5 (11.83)	9.84	40.3	4.95/56.87
SO_2_ (μg/m^3^)	6.57 (2.64)	3.37	9.92	1.5/21.9	.	.	.	.	19.46 (26.75)	4.3	53.2	0.9/232.4	10.5 (4.07)	5.53	15.9	1.5/27.5
O_3_ (μg/m^3^)	38.66 (13.47)	23.83	56.98	12.81/103.31	56.47 (16.5)	36.53	77.85	10.33/116.1	74.15 (24.38)	39.8	104.6	9.7/136.8	48.31 (13.92)	32.53	66.22	9.77/115.1
PM_10_ (μg/m^3^)	49.8 (15.54)	31	71.45	16/171.92	51.15 (13.8)	34.67	69	18/146	32.43 (12.79)	15.5	48.5	4/78.5	44.46 (9.55)	32.8	57.55	19.84/83.4
PM_2.5_ (μg/m^3^)	27.06 (10.54)	14	41	7/75	26.01 (7.91)	16.5	37	9/68.5	15.71 (4.49)	10.33	21	5/49.33	20.32 (7.28)	12	29.5	3/50
Temperature (°C)	14.15 (0.71)	13.23	15.01	10.65/16.26	23.57 (1.17)	22.21	25.03	19.43/31.6	24.84 (1.34)	22.92	26.43	20.04/28.32	23.57 (1.17)	22.21	25.03	19.43/31.6
Relative humidity (%)	2.52 (4.13)	0	7.97	0/24.95	2.32 (6.74)	0	6.13	0/95.5	7.32 (17.89)	0	25.34	0/185.66	2.32 (6.74)	0	6.13	0/95.5
Precipitation (mm)	64.58 (5.02)	58.36	71.35	50.34/81.28	59.2 (25.22)	16.2	82.28	3.61/94.64	68.39 (6.94)	60.25	77.82	49.22/91.39	59.2 (25.22)	16.2	82.28	3.61/94.64

P10 = percentile 10; P90 = percentile 90

Missing data were excluded from the analysis. However, we did not used any imputation method as we obtained a validated database for air pollutants concentration from the National Government with daily pollutant concentration values for nearly all of the 1461 days in the time-series, with the exception of PM2.5 (3% of the series was missing data in Medellin, 7% in Bogota, 5% in Bucaramanga and Cali).

Table 2 presents the conditional, multi-city Poisson models for total suicides, and suicides by gender for each pollutant. At first, spurious statistically significant associations were found in the crude models (i.e., not adjusted by holidays or climatic variables) for NO_2,_ PM_10_, PM_2.5_ and CO. In contrast, the estimates that take into account the confounding factors suggest that none of the pollutants are significantly associated with total suicide count or suicide counts by sex (*p* > 0.05). Estimators adjusted by temperature, precipitation, relative humidity and holidays for total suicides obtained from the conditional Poisson model for each air pollutant were non statistically significant: NO_2_ (IRR:0.99, 95% CI: 0.95–1.04), SO_2_ (IRR:0.99, 95% CI: 0.98–1.01), PM_10_ (IRR:0.99, 95% CI:0.95–1.03), PM_2.5_ (IRR:1.01, 95% CI: 0.98–1.05), CO_Max_(IRR:1.00, 95% CI:1.00–1.00) and O_3_ (IRR: 1.00, 95% CI: 0.96–1.04).

**Table 2:** Conditional Poisson regression models for suicide counts among four Colombian cities, 2011–2014

Pollutant	Crude Estimations				Adjusted Estimations^a^			
	IRR	95% CI		p	IRR	95% CI		p
Models for total number of suicides								
NO_2_	0.82	0.80	0.85	< 0.01	0.99	0.95	1.04	0.81
SO_2_	1.00	0.99	1.01	0.52	0.99	0.98	1.01	0.31
PM_10_	0.96	0.93	0.99	< 0.01	0.99	0.95	1.03	0.64
PM_2.5_	0.95	0.92	0.98	< 0.01	1.01	0.98	1.05	0.47
CO_max_	0.99	0.99	0.99	< 0.01	1.00	1.00	1.00	0.81
O_3_	1.00	0.98	1.03	0.851	1.00	0.96	1.04	0.93
Models for suicides among men								
NO_2_	0.82	0.80	0.85	< 0.01	0.99	0.94	1.04	0.69
SO_2_	1.00	1.00	1.01	0.34	0.99	0.98	1.01	0.35
PM_10_	0.96	0.93	0.99	0.02	1.00	0.95	1.04	0.86
PM_2.5_	0.95	0.92	0.98	< 0.01	1.00	0.97	1.04	0.80
CO_max_	0.99	0.98	0.99	< 0.01	1.00	1.00	1.00	0.69
O_3_	1.01	0.98	1.03	0.65	1.00	0.96	1.04	0.93
Models for suicides among women								
NO_2_	0.82	0.76	0.88	< 0.01	1.01	0.92	1.12	0.77
SO_2_	0.99	0.97	1.02	0.64	0.99	0.96	1.03	0.69
PM_10_	0.95	0.88	1.02	0.18	0.97	0.88	1.06	0.47
PM_2.5_	0.94	0.88	1.01	0.08	1.00	0.93	1.09	0.92
CO_max_	0.99	0.98	0.99	< 0.01	1.00	0.99	1.01	0.77
O_3_	0.99	0.93	1.04	0.62	1.00	0.91	1.09	0.93

For each pollutant the IRR are mean changes in the rates per increase in this 20% of the average (6 μg/m^3^ for NO_2_, 2 μg/m^3^ for SO_2_, 0.4 μg/m3 for CO and 10 μg/m^3^ for O_3_). For PM_10_ and PM_2.5_, the values were centered by convention on 10 and 5 μg/m^3^, respectively

*IRR* incidence rate ratio, *CI* confidence interval

^a^Estimates are adjusted for temperature, precipitation, humidity and holidays

Similarly, these associations were not found to be statistically significant after stratifying by sex and age group (Table 3). Moreover, single day and 7-day cumulative lagged effects were not found statistically significant in men neither in women (Table 4).

**Table 3:** Conditional Poisson regression models for suicides among four Colombian cities stratified by sex and age group, 2011–2014

Sex	Pollutant	Children				Adults				Older adults			
		IRR	95% CI		p	IRR	95% CI		p	IRR	95% CI		p
Men	NO_2_	1.08	0.79	1.47	0.62	0.98	0.93	1.03	0.45	1.04	0.90	1.21	0.57
	SO_2_	0.70	0.53	1.04	0.16	0.99	0.98	1.00	0.39	1.00	0.82	1.35	0.68
	PM_10_	0.89	0.67	1.19	0.44	0.99	0.94	1.04	0.72	1.04	0.92	1.18	0.53
	PM_2.5_	0.97	0.76	1.23	0.79	1.00	0.96	1.04	0.96	1.14	1.02	1.28	0.02^a^
	CO_max_	1.00	0.98	1.03	0.62	1.00	1.00	1.00	0.45	1.00	0.99	1.01	0.57
	O_3_	0.91	0.70	1.19	0.51	1.00	0.96	1.06	0.88	0.99	0.88	1.12	0.90
Women	NO_2_	0.56	0.30	1.01	0.10	1.05	0.95	1.17	0.36	0.95	0.66	1.38	0.80
	SO_2_	0.66	0.39	1.14	0.14	1.00	0.96	1.04	0.96	0.93	0.79	1.09	0.38
	PM_10_	0.60	0.35	1.03	0.06	0.97	0.88	1.08	0.60	1.05	0.82	1.35	0.68
	PM_2.5_	0.79	0.53	1.16	0.23	1.00	0.92	1.09	0.97	1.17	0.96	1.43	0.13
	CO_max_	0.96	0.92	1.00	0.06	1.00	1.00	1.01	0.36	1.00	0.97	1.02	0.80
	O_3_	1.21	0.73	1.99	0.45	0.99	0.90	1.09	0.83	0.97	0.76	1.25	0.84

For each pollutant the IRR are mean changes in the rates per increase in this 20% of the average (6 μg/m^3^ for NO_2_, 2 μg/m^3^ for SO_2_, 0.4 μg/m3 for CO and 10 μg/m^3^ for O_3_). For PM_10_ and PM_2.5_, the values were centered by convention on 10 and 5 μg/m^3^, respectively

*IRR* incidence rate ratio, *CI* confidence interval

Estimates are adjusted for temperature, precipitation, humidity and holidays

^a^No significant considering Bonferroni correction

**Table 4:** Models with lagged and cumulative effects for suicides among four Colombian cities stratified by sex, 2011–2014

Pollutant	Men					Women			
	Lag	IRR	95% CI		p	IRR	95% CI		p
NO_2_	L0	0.98	0.92	1.05	0.61	0.98	0.86	1.12	0.79
	L1	0.96	0.89	1.03	0.25	1.00	0.86	1.17	0.96
	L2	1.02	0.95	1.10	0.61	0.97	0.83	1.13	0.69
	L3	1.05	0.98	1.14	0.18	1.05	0.90	1.22	0.57
	L4	1.06	0.98	1.14	0.16	1.18	1.01	1.37	0.03^a^
	L5	0.93	0.87	1.01	0.09	1.03	0.88	1.20	0.71
	L6	1.00	0.93	1.08	0.95	0.94	0.80	1.09	0.41
	L7	1.00	0.93	1.06	0.89	0.96	0.84	1.09	0.50
	L1–7	0.99	0.93	1.04	0.61	1.06	0.95	1.19	0.27
SO_2_	L0	1.02	0.97	1.07	0.51	0.96	0.85	1.08	0.47
	L1	0.95	0.89	1.01	0.07	0.96	0.84	1.09	0.52
	L2	0.97	0.92	1.03	0.35	0.98	0.86	1.11	0.73
	L3	1.05	0.99	1.11	0.09	1.05	0.92	1.19	0.50
	L4	0.98	0.92	1.04	0.49	1.06	0.93	1.21	0.36
	L5	1.03	0.98	1.09	0.26	0.99	0.86	1.13	0.83
	L6	0.96	0.91	1.02	0.18	0.97	0.85	1.11	0.65
	L7	1.01	0.96	1.06	0.71	0.96	0.85	1.08	0.47
	L1–7	1.00	0.98	1.00	0.15	0.99	0.96	1.03	0.63
PM_10_	L0	0.96	0.92	1.02	0.18	1.00	0.90	1.12	0.93
	L1	1.01	0.95	1.07	0.83	0.96	0.85	1.09	0.55
	L2	0.98	0.92	1.04	0.51	0.99	0.88	1.12	0.87
	L3	1.04	0.99	1.11	0.13	1.02	0.91	1.14	0.70
	L4	1.05	0.99	1.12	0.08	1.12	0.99	1.25	0.06^a^
	L5	0.98	0.92	1.04	0.43	0.92	0.82	1.05	0.22
	L6	0.99	0.93	1.05	0.68	1.00	0.88	1.13	0.94
	L7	0.99	0.94	1.04	0.72	0.98	0.88	1.09	0.67
	L1–7	0.99	0.94	1.05	0.82	0.99	0.88	1.11	0.83
PM_2.5_	L0	1.01	0.96	1.05	0.83	1.03	0.94	1.14	0.50
	L1	1.00	0.94	1.05	0.92	0.93	0.83	1.05	0.24
	L2	0.98	0.92	1.03	0.42	1.05	0.93	1.17	0.44
	L3	1.02	0.96	1.08	0.50	0.97	0.87	1.09	0.65
	L4	1.06	1.00	1.12	0.04^a^	1.16	1.04	1.29	0.01^a^
	L5	0.98	0.93	1.03	0.43	0.89	0.79	0.99	0.04
	L6	0.98	0.93	1.04	0.49	0.95	0.85	1.07	0.38
	L7	1.01	0.96	1.05	0.82	1.04	0.95	1.15	0.38
	L1–7	1.01	0.97	1.06	0.57	0.99	0.91	1.08	0.87
CO_max_	L0	1.00	0.99	1.00	0.61	1.00	0.99	1.01	0.79
	L1	1.00	0.99	1.00	0.25	1.00	0.99	1.01	0.96
	L2	1.00	1.00	1.01	0.61	1.00	0.99	1.01	0.69
	L3	1.00	1.00	1.01	0.18	1.00	0.99	1.01	0.57
	L4	1.00	1.00	1.01	0.16	1.01	1.00	1.02	0.03^a^
	L5	1.00	0.99	1.00	0.09	1.00	0.99	1.01	0.71
	L6	1.00	1.00	1.01	0.95	1.00	0.99	1.01	0.41
	L7	1.00	1.00	1.00	0.89	1.00	0.99	1.00	0.50
	L1–7	1.00	0.99	1.00	0.61	1.00	0.99	1.01	0.27
O_3_	L0	0.98	0.93	1.04	0.52	1.07	0.95	1.20	0.25
	L1	1.05	0.98	1.11	0.16	0.96	0.84	1.10	0.54
	L2	1.02	0.96	1.09	0.56	1.00	0.87	1.14	0.96
	L3	0.98	0.92	1.04	0.45	0.93	0.81	1.06	0.29
	L4	1.02	0.95	1.08	0.62	1.04	0.91	1.18	0.59
	L5	0.97	0.91	1.03	0.33	0.98	0.86	1.12	0.75
	L6	1.05	0.98	1.12	0.14	0.97	0.85	1.11	0.65
	L7	1.01	0.96	1.00	0.63	1.04	0.93	1.17	0.49
	L1–7	1.05	1.00	1.10	0.05	0.97	0.88	1.07	0.52

For each pollutant the IRR are mean changes in the rates per increase in this 20% of the average (6 μg/m^3^ for NO_2_, 2 μg/m^3^ for SO_2_, 0.4 μg/m3 for CO and 10 μg/m^3^ for O_3_). For PM_10_ and PM_2.5_, the values were centered by convention on 10 and 5 μg/m^3^, respectively. L1-L7 represent the cumulative effect of the moving average in the last 7 days

*IRR* incidence rate ratio, *CI* confidence interval

Estimates are adjusted for temperature, precipitation, humidity and their lags (from one day to up 7 days) as well as holidays

^a^No significant considering Bonferroni correction

## Discussion

In this study, we did not find any statistically significant association between daily air pollutant concentrations and the daily number of suicides in four Colombian cities. However, we did find significant “crude” associations when the preliminary data had not yet been adjusted for confounding factors, even when using a model that recognizes self-correlation of observations. Thus, these results suggest that some previous studies could have inaccurately reported positive associations when omitting important confounding factors, a possibility that has also been pointed out in other studies [18, 19].

The previous studies that reported a significant association between air pollution and suicide rate are relatively heterogeneous in terms of the air pollutants involved. For example, while none of the studies that evaluated the effects of CO [9, 11] found an association, three out of five studies examining NO_2_ [8, 10, 12] found an association, and only one study reported an association with SO_2_ [9]. In contrast, all three studies that evaluated the effects of O_3_ [9, 11, 28] consistently found an association. Furthermore, with respect to PM, five out of the six studies found an association (including PM_10_ and PM_2.5_) [9–12, 29]. In this study we did not found any association of suicide counts and air pollutants concentrations after controlling for meteorological factors and the influence of holidays.

In some of the previous studies, associations were only found within specific age groups [10]. Likewise, in the studies by Bakian et al. [12] and Kim et al. [29], an association was only found in men and individuals with cardiovascular disease, respectively. Here, although we did not specifically explore the effect on individuals with underlying health conditions, no differences in the results were found when examining the data by age group or gender. Furthermore, Lin et al. [10] reported that there could be an interaction between the different levels of pollution, the mixture of pollutants, and/or specific factors of the population, and that such interactions could explain why associations appear in some populations but not others. Thus, it would be interesting to further investigate such potential interactions in future studies.

It is important to note that our study has the typical limitations of ecological approaches and estimations and conclusions can be only interpreted at population level as individual heterogeneity is not taken into account. For example, although not explored in our study, individual susceptibility to air pollution or other conditions such as interactions with other allergens could have an impact on associations, which have been reported in other studies [22]. Moreover, related to the abovementioned limitation is the fact that analyses based on broad geographic areas such as cities (as is the case with most studies that analyze the effect of air pollution on health) do not consider relationships on a smaller scale. That is, our estimations are based on the city average of pollutant concentrations, meteorological variables and on the total daily suicide number. Despite the fact that there are an important number of air quality and meteorological stations across cities, they are not necessarily distributed randomly over the entire study areas, and thus a sociological fallacy could be incurred [30]. Therefore, as the results of this study are applied on a population level, they cannot be extrapolated to the individual or small-area level.

In Colombia, DANE official mortality records and verified and validate by experts in death codifications system and is estimated that accounts for approximately 95% of the deaths in urban areas. However, It is also important to consider the probability of misclassification and missing suicide data due to codification and diagnosis errors [31].

The present study serves to reduce the possible publication bias that can occur when researchers, reviewers and editors send or accept manuscripts based only on the strength or positivity of the findings [32]. Recognizing and preventing publication biased is an important task, both for the general scientific perspective (complete dissemination of knowledge) and for those who combine results from a number of similar studies (i.e., systematic reviews and meta-analyses) [32, 33]. Within this framework, our negative results therefore contribute to the available evidence regarding the relationship between pollution and suicide.

## Conclusions

After adjusting for major confounding factors, we found no statistically significant association between air pollution and suicide in the four major Colombian cities. These “negative” results provide further insight into the current discussion regarding the existence of such a relationship. Employing a robust statistic approach and taking into account the main confounding factors, our study provides high quality evidence that could help clarify the relationship between air pollution and suicide.

## Abbreviations

CO: Carbon monoxide
DANE: National Administrative Department of Statistics (Departamento Administrativo Nacional de Estadística)
ICD-10: International Classification of Diseases, tenth edition
IDEAM: Institute of Hydrology, Meteorology and Environmental Studies (Instituto de Hidrología, Meteorología y Estudios Ambientales)
MM: Millimeter
NO_2_: Nitrogen dioxide
O_3_: Ozone
PM: Particulate matter
PM_10_: Particulate matter 10 μm or less in diameter
PM_2.5_: Particulate matter 2.5 μm or less in diameter
SO_2_: Sulfur dioxide
VIT: Dispersion index
